# Supplementary material for: Ammonia and Humidity Sensing by Phthalocyanine–Corrole Complex Heterostructure Devices
Source: Sensors (Basel). 2023 Jul 28;23(15):6773. doi: 10.3390/s23156773 (PMC10422489; doi:10.3390/s23156773)
Supplement: Supplementary file 1 [file sensors-23-06773-s001.zip › sensors-2440457-supplementary.pdf]

Supporting Information

Ammonia and Humidity Sensing by Phthalocyanine-Corrole Complex Heterostructure Devices

Lorena Di Zazzo [1,2], Sujithkumar Ganesh Moorthy [1], Rita Meunier-Prest [1], Eric Lesniewska [3], Corrado Di Natale [2,*], Roberto Paolesse [4] and Marcel Bouvet [1,*]

Table S1. Characteristic Raman bands (cm^{-1}) of CuT(pCH_3O)PC, CuT(pF)PC and LuPc_2 complexes, compared to these of double layer heterojunctions.

Cu(pmethoxy)TPC Evaporated powder	MSDI Cu(pCH3O)TPC/LuPc_2	Cu(pF)TPC Evaporated powder	MSDI Cu(pF)TPC/LuPc_2	LuPc_2 powder	Assignment
	549.322			547 w	Pc breathing
	578.16			578.16 w	Pc breathing
		647.353	645.67		C-F [1,2]
657.67 w	660.8				C-C-H in benzene ring
	680.97			680	
	739.32		735.995	734	C-H wagging
780.813	782.468		780.813	779	C=N aza stretching
815.513					
851.73					
882.868	884.505				
980.507	982.125				
	1009.59	1016.03	1016.03	1011	C-H binding
1054.63	1054.63	1057.83	1056.23	1046	
1075.46	1075.46	1080.26	1080.126		
	1105.81			1103	C-H binding
	1121.75		1121.75	1121	
				1146	Pyrrole breathing
	1158.28			1160	
1177.28	1177.28	1177.28	1177.28	1176	C-H bending
1193.08	1196.24				
1221.45	1223.02			1217	C-H bending
1249.72					
1265.39		1263.82			
	1273.21				
1290.39	1291.95				
		1305.98	1304.43	1301	C-H bending
1312.12	1312.21				
1340.19	1343.29	1340.19	1337.08		Cα-Cα
1363.42 m			1363.42		Cα-Cα
1402 m	1406.63		1408.17	1406.63	Cα-Cmeso
		1441.96			Cα-Cα
1486.29 s	1487.81	1501.52			Cα-Cα
1518.24	1515.2	1522.8	1507.6	1508	Coupling of pyrrole and aza stretching
	1527.35		1528.87		
1601.35 m	1599.85		1601.35	1599.85	C=C in benzene ring

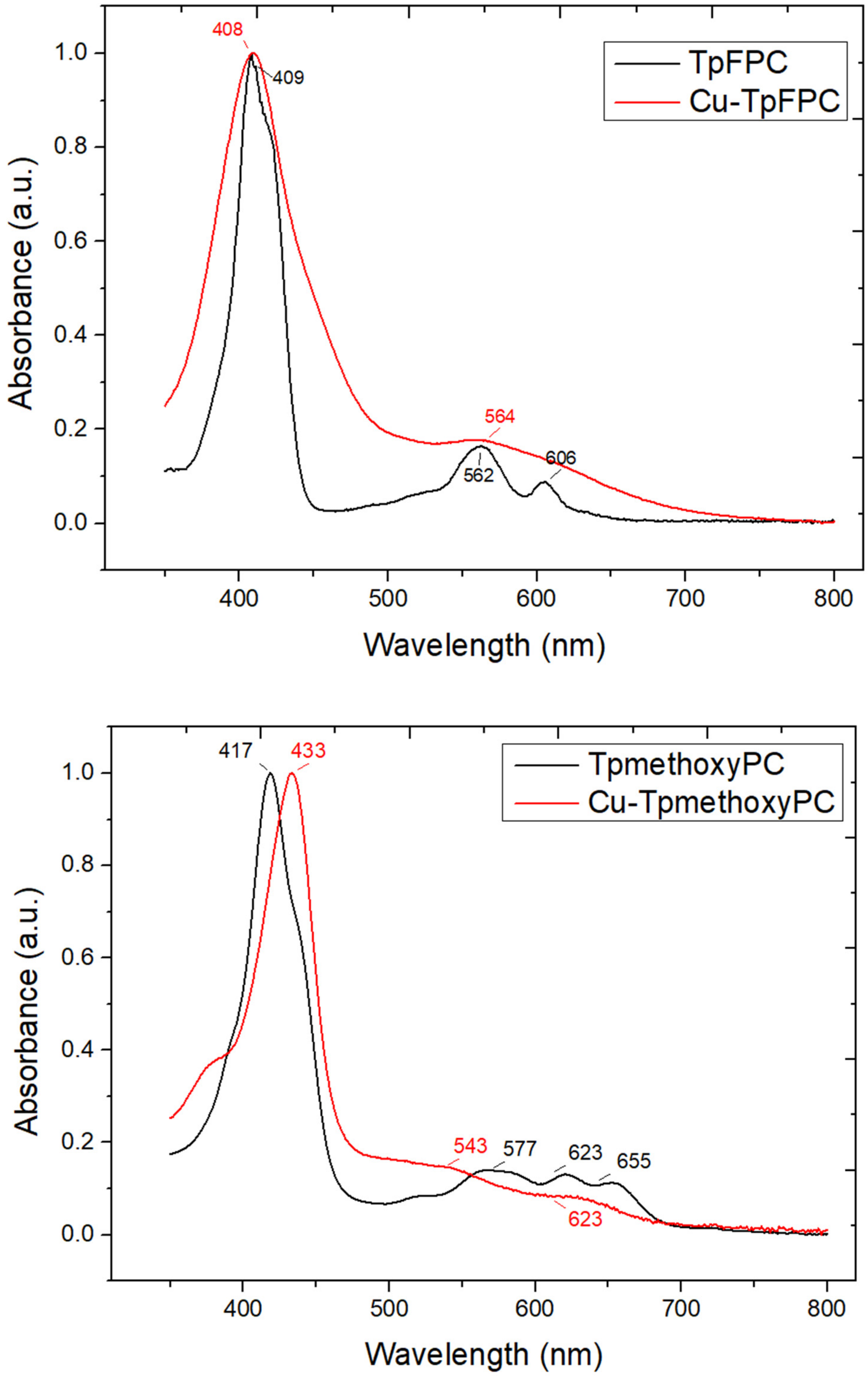

Figure S1. UV-visible electronic absorption spectra of **1** (a) and **2** (b) in $CHCl_3$ solution (black) compared to the corresponding metal free corroles (red).

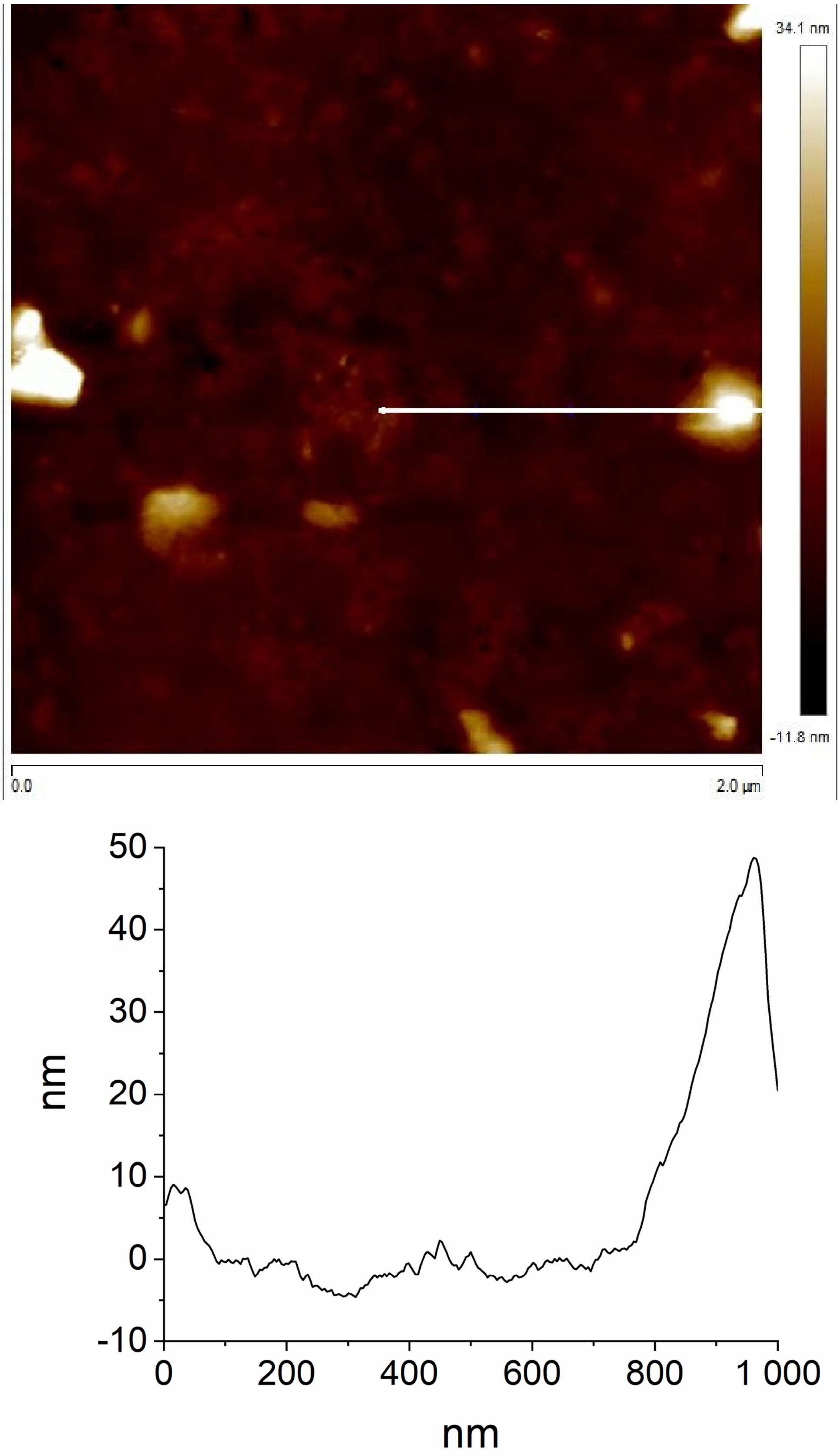

Figure S2. AFM images of a particular area of **2**/$LuPc_2$ device, from top to bottom: 2 µm x 2 µm 2D picture and the profile corresponding to the 1 µm-long line shown on 2D picture, respectively.

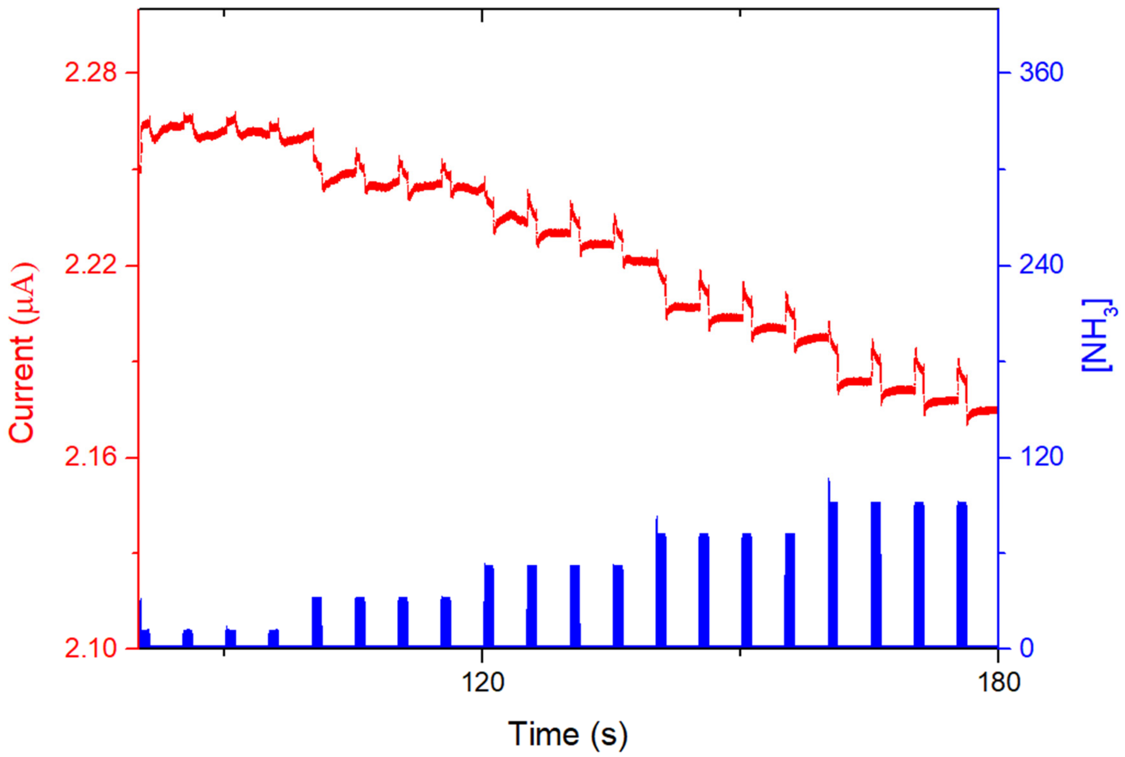

Figure S3. Current variation as a function of time of **1**/$LuPc_2$ heterojunction device exposed to NH_3 in the range 10-90 ppm with 1 min/4 min exposure/recovery cycles, at 40% RH and a bias of 3 V.

References

1. Sharts, C. M.; Gorelik, V. S.; Agoltsov, A. M.; Zlobina, L. I.; Sharts, O. N. Detection of carbon-fluorine bonds in organofluorine compounds by Raman spectroscopy using a copper-vapor laser. *Proc. SPIE-Int. Soc. Opt. Eng.* **1999**, *3537*, 317.
2. Menaa, F.; Menaa, B.; Sharts, O. Development of carbon-fluorine spectroscopy for pharmaceutical and biomedical applications. *Faraday Discussions* **2011**, *149*, 269.
